# Enrichment-Free Rare Cell Profiling in Pediatric Osteosarcoma Reveals a Circulating Cell Population Associated with Relapse-Free Survival

**DOI:** 10.3390/cancers18193123

**Published:** 2026-09-26

**Authors:** Stephanie N. Shishido, Siddhant Chaudhary, Ian Ward, Camilla Plascencia-Laija, Anya Zdanowicz, Venkata Yellapantula, Jeremy Mason, James Hicks, Fariba Navid, Peter Kuhn

**Affiliations:** 1 Convergent Science Institute in Cancer (CSI-Cancer), Michelson Center for Convergent Bioscience, University of Southern California, Los Angeles, CA 90089, USA; sshishid@usc.edu (S.N.S.); sschaudh@usc.edu (S.C.); ivward@usc.edu (I.W.); camillaplascencialaija@gmail.com (C.P.-L.); masonj@usc.edu (J.M.); jameshic@usc.edu (J.H.); 2 Cancer and Blood Disease Institute, Children’s Hospital Los Angeles, Los Angeles, CA 90027, USA; azdanowicz@chla.usc.edu (A.Z.); vyellapantula@chla.usc.edu (V.Y.);; 3 Department of Pediatrics, Keck School of Medicine, University of Southern California, Los Angeles, CA 90027, USA; 4 Division of Genomic Medicine, Department of Pathology and Laboratory Medicine, Children’s Hospital Los Angeles, Los Angeles, CA 90027, USA; 5 USC Norris Comprehensive Cancer Center, Keck School of Medicine, University of Southern California, Los Angeles, CA 90033, USA; 6 Department of Biological Sciences, Dornsife College of Letters, Arts and Sciences, University of Southern California, Los Angeles, CA 90089, USA; 7 Catherine and Joseph Aresty Department of Urology, Institute of Urology, Keck School of Medicine, University of Southern California, Los Angeles, CA 90033, USA; 8 Department of Aerospace and Mechanical Engineering, Viterbi School of Engineering, University of Southern California, Los Angeles, CA 90089, USA; 9 Department of Biomedical Engineering, Viterbi School of Engineering, University of Southern California, Los Angeles, CA 90089, USA

**Keywords:** osteosarcoma, circulating tumor cells, liquid biopsy, DAPI-only, non-enrichment, outlier detection, deep learning, pediatric sarcoma, Kaplan-Meier, prognostic biomarker, relapse-free survival, vimentin, copy number alteration

## Abstract

Background: Outcomes for patients with osteosarcoma (OS) presenting with metastases at diagnosis or relapsed remain poor with five-year survival rate of only 20–30%. Risk stratification at diagnosis remains limited, and no validated prognostic liquid biopsy analyte currently exists for this disease. OS lacks the recurring mutational hotspots and epithelial surface markers that underpin liquid biopsy strategies developed for carcinomas, necessitating fundamentally different approaches to circulating biomarker detection. Methods: Peripheral blood samples from 25 pediatric and young adult OS patients at clinically apparent disease timepoints (*n* = 29 samples) and 76 adult healthy donors (ND) were processed without cell enrichment using fluorescent whole-slide imaging. An outlier detection pipeline employing deep contrastive learned features classified rare cells across eight immunophenotypic categories defined by DAPI, vimentin (V), cytokeratin (CK), and CD45/CD31 (CD) expression. From the same patient cohort, plasma cell-free DNA (cfDNA) was analyzed by ichorCNA-based tumor fraction estimation across 42 samples (10 clinically apparent disease timepoints shared with the rare cell cohort and 32 alternative timepoints) from ten patients, and single-cell copy number alteration (scCNA) profiling was performed on 80 rare cells from five patients. Results: Rare cells were detected at a median of approximately 127 cells/mL (range: 29–1114) in OS patients at clinically apparent disease timepoints versus 34 cells/mL (range: 4–225) in healthy donors. DAPI-only (D) cells, identified using a data-driven optimal cutpoint (38.32 cells/mL) that is subject to optimization bias and requires independent validation, were associated with relapse-free survival in this exploratory, single-center OS cohort (log-rank *p* = 0.0003), with high D cell burden associated with markedly shorter relapse-free survival (median 393 days, 95% CI: 106–572) compared to patients with low D cell counts (median 835 days, 95% CI: 494–1671). D cells also showed the broadest clinical associations, correlating with metastatic burden and disease subgroup. The Vimentin^+^/CD45^−^ (D|V) population was the strongest discriminator between OS patients and healthy donors (*p* = 4.64 × 10^−12^) but did not distinguish relapse-free survival groups within the OS cohort (log-rank *p* = 0.8367). Genomic analyses at the plasma, single-cell, and tumor tissue levels were largely uninformative. Conclusions: Enrichment-free rare cell profiling identifies a DAPI-only circulating rare cell population whose abundance is associated with relapse-free survival in this exploratory, single-center OS cohort, accessible by nuclear morphology alone without biomarker enrichment. This is a novel, hypothesis-generating observation that, together with the mesenchymal-forward D|V population characterized here, motivates a larger, independent, prospective validation cohort and dedicated single-cell molecular (proteomic) characterization of the D-cell population, rather than establishing DAPI-only rare cell enumeration as a validated liquid biopsy stratification tool at this stage.

## Simple Summary

Osteosarcoma is the most common bone cancer in children and teenagers, and outcomes for patients whose cancer spreads or returns after treatment have not improved in decades. A major barrier to progress is the lack of tools that can help identify, at the time of diagnosis, which patients are most likely to relapse. In this exploratory study, we analyzed blood samples from 25 pediatric osteosarcoma patients using an approach that scans every cell in the sample without pre-selecting for any particular marker. We identified a population of rare circulating cells, distinguishable by their size and nuclear shape alone, whose abundance in the blood at the time of active disease was associated with how quickly patients relapsed. Patients with high counts of these cells relapsed sooner than those with low counts. This observation suggests that a simple blood-based cell count, requiring no specialized capture technology, may help predict relapse risk at diagnosis, but it will need to be confirmed in a larger, independent patient cohort before any clinical use can be considered.

## 1. Introduction

Among malignant bone tumors affecting children, adolescents, and young adults, osteosarcoma (OS) is the most prevalent, accounting for approximately 60% of primary bone malignancies in this age group [1]. Patients with localized disease at diagnosis treated with multi-agent systemic therapy combined with surgical resection have a 5-year survival rate of 60–70%. However, outcomes for patients who relapse or present with metastases at diagnosis remain dismal, 5-year survival rate of 20–30% [2]. A critical barrier to progress is the absence of validated, minimally invasive tools for risk stratification at diagnosis and disease monitoring during treatment. Identifying which patients harbor occult aggressive disease before metastasis is radiographically apparent remains an unmet clinical need, a gap recently reviewed across primary bone malignancies [3].

Liquid biopsy offers a conceptually attractive approach to this problem, but the two dominant strategies developed for carcinomas translate poorly to OS. EpCAM-based circulating tumor cell (CTC) enrichment depends on a marker that is expressed at variable and generally low levels in sarcoma [4], making the FDA-cleared CellSearch platform biologically inappropriate for this tumor type. ctDNA-based assays face a different constraint: OS is defined genomically by structural rearrangements, chromothripsis, and large-segment copy number changes [5] rather than the hotspot point mutations that enable sensitive ctDNA detection, limiting detectable tumor fractions in routine blood draws [6,7].

Prior efforts to detect sarcoma CTCs using alternative strategies have demonstrated feasibility but not clinical utility at the level of individual patient stratification [8,9]. Size-based filtration [10], cell surface vimentin (CSV) immunoaffinity capture [11,12], and ganglioside-targeting microfluidic devices [13] have each enriched mesenchymal rare cells from sarcoma patient blood. High-multiplexed protein profiling of CSV-captured OS CTCs has confirmed inter-patient heterogeneity [14]. CTC burden and phenotype have additionally been associated with clinicopathological stage, treatment response, and relapse risk in independent osteosarcoma cohorts [15,16,17,18]. Separately, EMT-marker CTC classification has associated elevated mesenchymal fractions with higher disease stage [8,19]. Collectively, this work establishes proof of concept but has not produced a clinically validated prognostic liquid biopsy analyte for OS.

An enrichment-free approach that processes all nucleated cells without pre-analytic selection, using automated fluorescence whole-slide imaging paired with an outlier detection pipeline employing deep contrastive learned features, offers a fundamentally different vantage point [20,21,22,23,24,25]. Rather than selecting for a specific surface antigen, this architecture identifies rare events computationally by their morphometric and immunophenotypic deviation from the dominant white blood cell population, preserving the full phenotypic breadth of circulating rare cells including mesenchymal-forward populations. This workflow has been validated across multiple solid tumor types [21,23,24,25]; this study represents its first systematic application to OS. We hypothesized that specific circulating rare cell populations detectable without enrichment will associate with clinically meaningful outcomes in OS, providing a minimally invasive prognostic signal not accessible through genomic liquid biopsy approaches.

## 2. Materials and Methods

### 2.1. Study Design and Patient Cohort

Peripheral blood was collected from 29 patients, ages 7–24 years, diagnosed with OS at Children’s Hospital Los Angeles (CHLA). For the primary analysis, 29 samples were selected from 25 of these patients, each drawn at a timepoint of clinically apparent disease, defined as a sampling date coinciding with confirmed diagnosis, disease progression, or relapse. Of these 29 samples, 24 were collected at pre-treatment baseline and 5 at disease progression timepoints. Among the 25 analyzed patients, 5 remained disease-free following standard therapy; 9 presented initially with localized disease and subsequently experienced relapse or treatment-refractory disease; and 11 presented with metastatic disease at diagnosis who later relapsed or were refractory to treatment. A concurrent cohort of 76 adult healthy donors (ND) provided peripheral blood as negative controls. The healthy donor cohort consisted of adults; this age difference is an acknowledged limitation when interpreting OS-vs-ND signal. All participants provided written informed consent (or parental/guardian consent for minors) under IRB-approved protocol IRB UP-24–00247 at CHLA and USC.

### 2.2. Blood Collection and Slide Preparation

Eight milliliters of peripheral blood was collected into cell-free DNA preservation tubes (Streck, La Vista, NE, USA) at each timepoint. White blood cell (WBC) counts were measured on arrival using a Medonic M-Series Hematology Analyzer (Clinical Diagnostic Solutions, Fort Lauderdale, FL, USA). Erythrocytes were removed by ammonium chloride lysis; the remaining nucleated cell fraction was washed, counted, and deposited at 3.5 × 10^6^ cells per custom-format glass slide (Marienfeld, Lauda-Königshofen, Germany). Slides were allowed to settle at 37 °C for 40 min, then blocked with 7% bovine serum albumin, air-dried, and stored at −80 °C until staining. Prior to staining, slides were equilibrated to room temperature for 2 h [20,21,22,23,24,25].

### 2.3. Immunofluorescence Staining

Staining was automated using the IntelliPATH FLX autostainer (Biocare Medical, Pacheco, CA, USA). After blocking with 10% goat serum, slides were incubated for 4 h with combinations of anti-human CD31:Alexa Fluor 647 (clone WM59, BioRad, Hercules, CA, USA; 2.5 μg/mL) and anti-mouse IgG goat Fab fragment (Jackson ImmunoResearch, West Grove, PA, USA; 100 μg/mL). Cells were then permeabilized with cold methanol (100%, 5 min). A 2-h antibody incubation included: pan-cytokeratin mixture targeting CK 1, 4, 5, 6, 8, 10, 13, 18, and 19 (clones C-11, PCK-26, CY-90, KS-1A3, M20, A53-B/A2, Sigma-Aldrich, St. Louis, MO, USA; C2562; 210 μg/mL); anti-CK19 (clone RCK108, Dako, Glostrup, Denmark; GA61561-2; 0.2 μg/mL); anti-CD45:Alexa Fluor 647 (clone F10-89-4, AbD Serotec, Hercules, CA, USA; MCA87A647; MCA87A647; 1.2 μg/mL); and anti-vimentin:Alexa Fluor 488 (clone D21H3, Cell Signaling Technology, Danvers, MA, USA; 9854BC; 3.5 μg/mL). Anti-mouse IgG1:Alexa Fluor 555 (Invitrogen, Carlsbad, CA, USA; A21127; 2 μg/mL) was applied for 1 h. Nuclei were counterstained with DAPI (1:50,000; Thermo Fisher Scientific, Waltham, MA, USA; D1306). Slides were mounted and sealed. Every run incorporated positive and negative controls.

### 2.4. Outlier Detection Pipeline with Learned Features

Stained slides underwent automated whole-slide scanning at 100× magnification across four fluorescence channels, producing 2304 imaging frames per slide (9216 total frames per scan). Rare cell identification and classification were performed using the OCULAR (Outlier Clustering Unsupervised Learning Automated Report) outlier detection pipeline, as previously described [21,22,23,24,25]. Briefly, the EBImage package in R (v4.22.1; R Foundation for Statistical Computing, Vienna, Austria) segments every DAPI-positive event and extracts 761 quantitative morphometric features per cell, spanning nuclear geometry, channel intensity, and pairwise channel relationships. A supervised random forest classifier identifies biologically relevant rare cell candidates, and three independent random forest models assign predicted expression levels for CK, V, and CD to each retained event, stratifying cells into eight immunophenotypic categories: D (DAPI-only), D|V, D|CD, D|V|CD, D|CK, D|CK|V, D|CK|CD, and D|CK|V|CD. A deep contrastive learning framework processes each cell image and generates 128 learned embeddings that capture morphometric and fluorescence properties in a compact representation space [22]. UMAP dimensionality reduction and Leiden clustering were applied to the same embedding space for unsupervised visualization and substructure analysis, as described in Section 2.8. All retained events underwent manual data curation by two trained analysts. Counts are expressed as events per mL.

### 2.5. Single-Cell Isolation and CNA Profiling

Individual cells were captured by an Eppendorf TransferMan NK2 micromanipulator (Eppendorf SE, Hamburg, Germany) and transferred into PCR tubes containing Tris-EDTA buffer, then frozen at −80 °C until amplification [21,23]. Whole-genome amplification (WGA) used the WGA4 Genomeplex kit (Sigma-Aldrich, St. Louis, MO, USA) with 23 PCR cycles. DNA was purified with QIAquick (Qiagen GmbH, Hilden, Germany), quantified by Qubit Fluorometer (dsDNA HS Assay, Thermo Fisher Scientific, Waltham, MA, USA), and sheared to approximately 200 bp (Covaris S2/E210; Covaris, Woburn, MA, USA). Libraries were prepared with NEBNext Ultra DNA Library Prep (New England Biolabs, Ipswich, MA, USA; E7370L) and sequenced on an Illumina NextSeq 500 or HiSeq2500 SR50 (Illumina, San Diego, CA, USA). Reads were trimmed, aligned to hg19 (Bowtie), sorted and deduplicated (SAMtools; Wellcome Sanger Institute, Hinxton, UK), and binned across 5000 UCSC genome windows. Circular binary segmentation (DNAcopy_1.26.0; Bioconductor; R Foundation for Statistical Computing, Vienna, Austria) generated genome-wide CNA profiles. Matched white blood cells (WBCs) served as co-processed controls; cells labeled as WBCs in the heatmap represent cells from the WBC fraction that were profiled alongside rare cells. Cells with fewer than 400,000 mapped bins were excluded from analysis to ensure sufficient read depth for reliable CNA calls, yielding 80 rare cells and 9 WBC controls across the five patients. Per-cell genomic instability (GI) scores were calculated using a ratio-to-median formulation with a hyperbolic tangent function [21]. The cfDNA and scCNA analyses were performed on distinct patient samples with no overlap between the two datasets.

### 2.6. Cell-Free DNA Analysis

Plasma cfDNA was extracted from 42 OS patient samples (10 clinically apparent disease timepoints shared with the rare cell analysis cohort and 32 additional longitudinal timepoints) drawn from the same patient cohort as the cell phenotyping analysis. Tumor fraction was estimated using the ichorCNA algorithm (Broad Institute, Cambridge, MA, USA) applied to ultra-low-pass whole-genome sequencing (ULP-WGS) data, following previously established methods [6,7]. A tumor fraction threshold of >10% was used as the validated confidence cutoff [6,7]. Fragment size distributions were characterized across three ranges: short (90–150 bp), mononucleosomal (140–190 bp), and dinucleosomal (300–380 bp).

### 2.7. Tumor Tissue Genomics

Diagnostic tumor tissue from a subset of patients was analyzed using the OncoKids^®^ Cancer Panel (Children’s Hospital Los Angeles Center for Personalized Medicine, CLIA/CAP-certified), a targeted panel covering hotspot mutations in 82 genes and amplifications in 24 genes [7]. Data were available for 21 of the 25 analyzed patients. Variant calls were integrated with clinical annotations.

### 2.8. Statistical Analysis

OS vs. ND comparisons used two-sided Wilcoxon rank-sum tests (*p* < 0.05) across the full panel of rare-cell phenotype and aggregate cell categories; the tissue-genomic and clinical correlation analyses likewise comprised multiple comparisons within their respective families. With the exception of the D and D|V survival analyses, which were specified a priori as the two strongest OS-vs-ND discriminators, all analyses in this study are exploratory and post hoc. Benjamini-Hochberg false discovery rate-adjusted Q values, computed across the complete set of comparisons within each family, are reported alongside nominal *p* values in the revised Tables 2–4. Rare cell counts were log-transformed for visualization. Associations between rare cell phenotypes and clinical or genomic variables used Wilcoxon rank-sum (categorical) or Spearman rank correlation (continuous). Normality for continuous variables was calculated using the Shapiro-Wilk Test. UMAP was applied to Z-normalized morphometric embeddings (*n* = 3626 cells); Leiden clustering was performed at a resolution of 0.002 to define phenotypic substructure. GI score comparisons used one-way ANOVA and Pearson correlation. Relapse-free survival (RFS) was estimated using the Kaplan-Meier method and compared by log-rank test; six patients were excluded from survival analyses due to insufficient follow-up time (defined as a total observation period of fewer than 90 days from diagnosis, or censoring prior to any event within the first 90-day landmark window). For D|V cells, patients were dichotomized at the cohort median (38.54 cells/mL), a standard and assumption-free cutpoint appropriate for the a priori hypothesis that D|V abundance separates patients. For DAPI-only (D) cells, a data-driven optimal binary cutpoint was identified using the maxstat algorithm (R package maxstat), which selects the threshold in a continuous variable that maximizes the standardized log-rank statistic for group separation in survival analysis [26,27]; the identified cutpoint was 38.32 cells/mL. This approach introduces optimization bias; the resulting threshold is hypothesis-generating and requires prospective validation. Fragment size comparisons used Mann-Whitney U tests with Benjamini-Hochberg correction. Analyses used R (v4.0.3+).

## 3. Results

### 3.1. Patient Cohort

This study draws on three distinct, partially-overlapping patient/sample cohorts: a rare-cell phenotyping cohort (29 samples from 25 patients, all at clinically apparent disease timepoints), a cell-free DNA (cfDNA) cohort (42 samples from 10 patients, comprising the 10 samples that overlap with the rare-cell cohort plus 32 additional longitudinal timepoints from the same 10 patients), and a single-cell copy-number alteration (scCNA) cohort (80 cells from 5 patients, entirely from the rare-cell phenotyping cohort, none from the cfDNA cohort); see Figure S1 for the complete sample-allocation schematic. Peripheral blood samples from 25 pediatric OS patients at clinically apparent disease timepoints (29 samples: 24 pre-treatment and 5 at disease progression) and 76 adult healthy donors (ND) were analyzed. Patient characteristics are provided in Table 1. Three clinical subgroups were defined: Group 1 (*n*  =  9), localized disease at diagnosis with subsequent relapse or treatment-refractory course; Group 2 (*n*  =  11), metastatic disease at diagnosis with subsequent relapse or refractory course; Group 3 (*n*  =  5), localized disease with sustained remission following standard therapy. A separate subset of 42 plasma samples from 10 patients (10 pre-treatment samples shared with the rare cell cohort and 32 alternative timepoints) was analyzed for cfDNA tumor fraction by ichorCNA-based ULP-WGS; 10 of these samples overlap with the clinically apparent disease timepoint cohort; the remaining 32 are alternative timepoints not used for rare cell enumeration. Sample allocation across analyte streams is summarized in Figure S1.

### 3.2. Circulating Rare Cell Detection: OS vs. Healthy Donors

#### 3.2.1. Rare Cells Are Significantly Elevated in OS Patients, with Multiple Phenotypic Populations Contributing to the Signal

Figure 1 presents a representative gallery of circulating rare cells detected in OS patient peripheral blood, illustrating the morphometric and immunophenotypic diversity of events captured by the enrichment-free workflow. Each row displays two independent examples of a single phenotypic class across five imaging channels (composite, DAPI, CK, V, and CD), highlighting the distinct nuclear morphology and staining patterns that define each category. DAPI-only (D) events, shown in row A, are notably larger than surrounding white blood cells and show no signal in the CK, V, or CD channels. D|V events (row C) display strong intracellular vimentin signal without CK or CD co-expression, consistent with a mesenchymal identity.

A heterogeneous mixture of circulating rare cells was detected across all OS patient samples. In OS patients, rare cells were detected at a median of 127.26 (range 29–1114) cells/mL, compared with a median of 33.57 cells/mL (range 4–225) in healthy donors (*p* = 2.60 × 10^−10^, Q = 8.65 × 10^−10^). Seven distinct rare cell populations reached statistical significance by Wilcoxon rank-sum test (Table 2; Figure 2). The two most strongly elevated populations were D|V (*p* = 4.64 × 10^−12^, Q = 4.64 × 10^−11^) and D (*p* = 1.88 × 10^−11^, Q = 9.38 × 10^−11^), with additional significant elevations in total cells, D|CD, D|V|CD, D|CK|V|CD, and total CK^+^ cells. The D|V population’s elevation relative to healthy donors is consistent with the mesenchymal identity of OS, as vimentin is constitutively expressed in OS tumor cells but absent from normal hematopoietic cells. However, vimentin expression is not exclusive to tumor-derived cells in circulation; as discussed further in Section 3.6, the primary prognostic content of the rare cell signal in this cohort derives from the D population rather than D|V.

The significant separation of total circulating rare cells and multiple phenotypic populations between OS patients and healthy donors establishes that the enrichment-free rare cell signal distinguishes patients with active OS from a normal peripheral blood background, a necessary prerequisite for clinical utility.

#### 3.2.2. Cell-Level UMAP and Leiden Clustering Reveal Spatially Organized Phenotypic Substructure

To characterize the morphometric and phenotypic organization of the rare cell landscape, we applied UMAP dimensionality reduction to the full Z-normalized morphometric embedding space from 3626 OCULAR-identified rare cells (Figure 3). Leiden clustering within this embedding identified 3 discrete spatial communities (Clusters 0–2, *n* ranging from 976 to 1370 cells per cluster). The three Leiden clusters showed distinct immunophenotypic compositions (Figure 3B). Cluster 0 (C0; *n* = 1370) was predominantly composed of D|V cells, with a smaller D|CD contribution, consistent with a mesenchymal-forward, CD45-negative rare cell population. Cluster 1 (C1; *n* = 1280) showed the most heterogeneous composition, with substantial representation across D|V|CD, D|CK|V|CD, D|CK|CD, and D|V populations, suggesting a mixed phenotypic neighborhood at the interface of mesenchymal and hematopoietic marker expression. Cluster 2 (C2; *n* = 976) was dominated by DAPI-only (D) cells, with minor contributions from CK-positive subpopulations, reflecting a biomarker-negative population distinguished primarily by nuclear morphology. Together, these cluster-phenotype relationships confirm that the UMAP embedding organizes rare cells along biologically coherent axes, with mesenchymal marker expression and morphometric features jointly shaping spatial position within the embedding.

Sex distribution across the UMAP embedding showed no spatially organized pattern, with male and female cells intermingled across all three major regions (Figure 3C), suggesting that morphometric and immunophenotypic variation in circulating rare cells is not sex-driven in this cohort. Blood lactate dehydrogenase (LDH) levels in patients showed a more structured distribution, with abnormal LDH concentrated in the upper and left regions of the embedding while normal LDH cells were more broadly distributed (Figure 3D), consistent with elevated tumor burden associated with specific rare cell morphometric states. Metastatic disease at diagnosis showed a similar spatial enrichment, with metastatic-origin cells predominating across the upper UMAP region and localized-disease cells more concentrated in the lower cluster (Figure 3E), supporting that disease extent at diagnosis is reflected in the morphometric properties of circulating rare cells.

Among continuous clinical variables, patient age showed no coherent spatial gradient across the embedding (Figure 3F), consistent with the narrow pediatric age range of this cohort limiting age-associated morphometric signal. Blood alkaline phosphatase (ALP) levels in patients displayed a gradient of elevated values concentrated in the upper UMAP region (Figure 3G), paralleling the LDH pattern and further supporting that systemic markers of bone turnover and tumor burden co-localize with specific morphometric rare cell states. Days to relapse showed the most spatially organized continuous gradient, with shorter relapse intervals concentrated in the upper and left regions of the embedding and longer intervals in the lower cluster (Figure 3H), suggesting that cells from patients with more aggressive relapse trajectories occupy a morphometrically distinct space within the rare cell landscape.

Individual patient contributions to the UMAP embedding were heterogeneous; the three highest-contributing patients (Patient 13: 727 cells, 20.0%; Patient 6: 348 cells, 9.6%; Patient 26: 235 cells, 6.5%) each localized preferentially to distinct Leiden clusters, confirming that the observed cluster structure reflects shared immunophenotypic properties across patients rather than patient-specific distributional artifacts (Figure S2).

The combined embedding of all 8055 OS and ND rare cells, colored by cohort and patient, is also provided for reference (Figure S3).

### 3.3. Genomic Analyses: cfDNA and Single-Cell CNA Profiling

#### 3.3.1. cfDNA Tumor Fraction Is Below the Detection Threshold in the Majority of Samples

Among 42 OS plasma samples (10 clinically apparent disease timepoints shared with the rare cell analysis cohort and 32 alternative timepoints) assessed by ichorCNA-based ULP-WGS, one sample exceeded the validated 10% tumor fraction confidence threshold (Patient 6; TF = 0.246; Figure 4A). Eight additional samples returned estimates between 3% and 10%, yielding 9 samples (21.4%) with detectable signal above the 3% detection limit. The remaining 33 samples (78.6%) fell at or below the detection limit. Fragment size analysis showed no significant differences between ichorCNA-passing (*n* = 9) and -failing (*n* = 33) samples across short (*p* = 0.208, FDR = 0.356), mononucleosomal (*p* = 0.231, FDR = 0.356), or dinucleosomal (*p* = 0.220, FDR = 0.356) fragment size ranges (Figure S4), confirming that the low informative yield reflects biological signal limitation rather than sample quality failure [6,7].

As an additional exploratory check of concordance between the two liquid biopsy analytes, we compared D-cell counts with cfDNA-derived (ichorCNA) tumor fraction at matched baseline timepoints for the 10 patients with both analytes available. D-cell count showed a positive but non-significant correlation with cfDNA tumor fraction (Pearson r = 0.61, R^2^ = 0.37, 95% CI −0.04 to 0.89, *p* = 0.064), consistent with a possible shared relationship to tumor burden that does not reach significance at this sample size (Figure S5).

#### 3.3.2. Single-Cell CNA Profiling: Classification-Associated Genomic Instability Signal Across Five Patients

Eighty rare cells from five OS patients (Patient 17, Patient 18, Patient 22, Patient 24 [relapse], and Patient 4 [progression]) passed the minimum bin count threshold of 400,000 mapped bins and were included in scCNA profiling alongside 9 matched WBC controls (Figure 5 and Figure S5); an additional 9 cells from the initial dataset of 98 were excluded for insufficient read depth. One additional patient was assessed but yielded only one recoverable cell and was excluded from analysis. These patients are distinct from those contributing cfDNA samples, with no overlap between datasets. CNA heatmaps revealed predominantly neutral profiles across all five patients, with no recurrent alterations meeting the clonality criterion of two or more cells sharing two or more discrete genomic alterations. The heatmap color scale is set such that values of 1.5 and 0.5 represent a gain or loss of one copy in a diploid cell; pale colors indicate less than a full copy change. One focal full loss (panel B, chromosome 5) and one focal full gain (panel C, chromosome 12) were observed as isolated single-cell events. Per-cell GI scoring by classification category revealed a statistically significant difference across groups (one-way ANOVA: F(6,73) = 3.67, η^2^ = 0.232, *p* = 0.0031), indicating that approximately 23.2% of the variance in GI scores is attributable to phenotypic classification. DAPI-only (D) cells showed the highest mean GI scores (mean = 182.3), followed by D|CD (mean = 120.6), while D|V cells showed intermediate instability (mean = 72.1). Pearson correlation between the number of expressed channel markers and per-cell GI score was weak and non-significant (r = −0.148, *p* = 0.190), indicating that phenotypic complexity per se does not linearly predict GI; rather, specific classification categories, particularly those lacking vimentin or cytokeratin expression, show elevated GI. These findings suggest that immunophenotypically simpler populations may harbor greater genomic disruption, warranting further investigation with deeper single-cell sequencing. Notably, CD45+ WBC-classified cells did not display the flat diploid profiles expected of leukocyte controls; their GI scores spanned a wide range (13.1–294.4; mean = 128.0) and were numerically higher than the rare cell population overall (mean = 88.6), though this difference did not reach statistical significance (Mann–Whitney U, *p* = 0.098). No recurrent copy-number alteration met our pre-specified clonality criterion (≥2 cells sharing ≥2 discrete alterations), and the elevated GI scores noted above did not resolve into a consistent clonal pattern. Given the well-documented technical noise inherent to single-cell whole-genome amplification, including replication-timing-associated copy-number artifacts described in S-phase single cells [28], we interpret these single-cell genomic findings as inconclusive rather than as evidence of a defined genomic alteration; resolving their biological significance was outside the scope of this study and is considered further in the Section 4.

### 3.4. Tumor Tissue Genomics and Tissue-to-Blood Correlations

Targeted genomic sequencing data (OncoKids^®^) were available for 21 of the 25 analyzed OS patients. The most frequently altered genes were TP53 (38%; 8/21), CCND3 (14.2%; 3/21), and CDK4 (9.7%; 2/21); 33.3% (7/21) of sequenced cases harbored no variants meeting clinical significance thresholds. Correlation analyses between tumor tissue genomic variants and circulating rare cell phenotypes revealed biologically plausible but clinically limited associations (Table 3). CCND3 and CDK4 alterations associated with D|V and D|CK|V populations, suggesting a potential link between cell-cycle dysregulation and mesenchymal circulating phenotype. None of these associations survived Benjamini-Hochberg correction for the 80 comparisons tested (Q ≥ 0.073; Table 3), and they should be interpreted as exploratory, hypothesis-generating signals rather than confirmed associations. TP53 alterations, the most prevalent event, did not correlated with rare cell counts. That 33.3% of patients harbored no clinically significant variants reflects the inadequacy of mutation-centric panels for a tumor type defined by structural rearrangement rather than hotspot mutation [5]. Tumor tissue genomic profiling in this cohort did not resolve patients by prognosis or identify a molecular subgroup with distinct clinical behavior.

### 3.5. Clinical Correlations with Circulating Rare Cells

Across all clinical correlation analyses, the DAPI-only (D) population emerged as the most frequently and broadly correlated analyte with disease-relevant features (Table 4). Days to relapse correlated with D cells (*p* = 0.005, Q = 0.443) and with total cells (*p* = 0.020, Q = 0.625). Metastases at diagnosis correlated with D (*p* = 0.006, Q = 0.443 [lung]; *p* = 0.015, Q = 0.625 [bone]). The D|V population showed more selective association with patient relapse status (*p* = 0.031, Q = 0.729). None of these clinical associations survived Benjamini-Hochberg correction for the 140 comparisons tested (Q ≥ 0.443; Table 4), and they should be interpreted as exploratory, hypothesis-generating observations rather than statistically confirmed findings.

### 3.6. DAPI-Only Circulating Rare Cells Stratify Relapse-Free Survival; D|V Cells Discriminate OS from Healthy Donors but Do Not Stratify Survival

#### 3.6.1. D Cells: Significant Prognostic Stratification of RFS

To evaluate whether circulating rare cell populations carry prognostic information, we performed Kaplan-Meier analysis of RFS using D and D|V cell counts as stratifying analytes in the 19 patients with sufficient follow-up time for survival analysis (six patients were excluded due to insufficient follow-up; Figure 6). D cell counts demonstrated significant stratification of RFS using a data-driven optimal cutpoint (38.32 cells/mL; Figure 6A). Patients with high D cell counts (*n* = 7, relapse events = 6) showed markedly shorter RFS (median 393 days, 95% CI: 106–572) compared to patients with low D cell counts (*n* = 12, relapse events = 12; median 835 days, 95% CI: 494–1671; log-rank *p* = 0.0003). The Kaplan-Meier curves separated sharply within the first 250 days from diagnosis: patients in the high D group experienced rapid early relapse events, with RFS probability dropping below 0.2 by approximately day 400; all seven high-D patients experienced a relapse event by day 600. By contrast, in the low D group, six patients remained relapse-free beyond day 750.

The DAPI-only rare cell count is analytically accessible: D cells are identifiable by nuclear morphology alone without requiring positive biomarker expression, representing the most analytically robust population in the enrichment-free workflow. The RFS association of D cells, combined with their correlations with metastatic burden, time to relapse, and disease subgroup (Section 3.5), is consistent with a biological role for this population that warrants further investigation; we do not interpret this association, on its own, as establishing D cells as a validated circulating prognostic analyte. The data-driven cutpoint introduces optimization bias appropriate to an exploratory cohort. To address this, we applied a permutation test and 1000-iteration bootstrap resampling (1000 iterations) to the D-cell log-rank statistic: the association remained significant under both a permutation-adjusted *p* = 0.027 and a bootstrap optimism-corrected *p* = 0.003, and the cutpoint was stable across resampling (significant in 100% of bootstrap resamples and leave-one-out cross-validation folds). As a complementary sensitivity analysis, we fit a Cox proportional hazards model treating D-cell count as a continuous covariate, supported by the available event count (19 events); higher D-cell burden was associated with shorter RFS (HR = 1.39 per 10 cells/mL, 95% CI 1.06–1.84, *p* = 0.018; per-SD HR = 1.93, 95% CI 1.12–3.34, *p* = 0.018), with no evidence of proportional-hazards violation (Schoenfeld *p* = 0.089). Given the limited event count, this model was kept univariable rather than extended to a multivariable model, which would risk overfitting. A complementary Cox model incorporating continuous nuclear morphometric features (e.g., area, eccentricity) was not performed in this study and is planned as a future analysis. Independent prospective validation is required before any clinical application can be considered.

#### 3.6.2. D|V Cells: OS-vs-Healthy-Donor Discriminator Without Prognostic Stratification of RFS

For D|V cells, dichotomization at the cohort median (38.54 cells/mL) yielded high (*n* = 9, relapse events = 9) and low (*n* = 10, relapse events = 9) groups with indistinguishable RFS (log-rank *p* = 0.8367; Figure 6B). D|V abundance at a single overt-disease timepoint does not predict subsequent relapse trajectory in this cohort, despite being the strongest OS-vs-ND discriminator. This dissociation emphasizes that markers of disease presence are not interchangeable with markers of disease aggressiveness, and that the prognostic content of the rare cell signal in this cohort resides in the D population rather than the D|V population. To confirm this null result was not an artifact of the median-split choice, we additionally applied the same data-driven optimal cutpoint search used for D cells to D|V: the maxstat algorithm identified only an extreme two-patient split (naive log-rank *p* = 0.12), and this association did not survive correction (permutation-adjusted *p* = 0.69; bootstrap optimism-corrected *p* = 1.00), with the cutpoint stable in only 5% of leave-one-out cross-validation folds, in marked contrast to the 100% stability observed for the D-cell cutpoint. A complementary Cox model treating D|V count as a continuous covariate was likewise null (HR = 1.12 per SD, 95% CI 0.65–1.95, *p* = 0.68). Together, these sensitivity analyses confirm that D|V abundance does not carry prognostic information for RFS in this cohort under any cutpoint or modeling approach.

As a sensitivity analysis, we repeated this comparison using overall survival (OS) rather than RFS as the endpoint, with both populations dichotomized at their respective cohort medians in the same 19 patients (Figure S7). D cell counts showed a concordant trend toward shorter OS in the high-count group (median OS not reached in the low group vs. 2373 days in the high group; log-rank *p* = 0.0565), and D|V cells again did not stratify outcome (median OS 2702 days [low] vs. 1331 days [high]; log-rank *p* = 0.0782). These OS results parallel the RFS findings above and support D cell burden, rather than D|V positivity, as the more consistent prognostic signal in this cohort.

## 4. Discussion

The central finding of this study is that a morphologically defined circulating rare cell population, identifiable by nuclear staining alone without any biomarker enrichment or positive immunophenotypic selection, is associated with relapse-free survival in this exploratory, single-center pediatric OS cohort (log-rank *p* = 0.0003). We regard this as a novel, hypothesis-generating observation that warrants confirmation in a larger, independent, prospective cohort together with dedicated molecular characterization of this population, rather than as evidence of a validated prognostic marker. Patients with high DAPI-only (D) cell counts at overt disease timepoints experienced markedly shorter RFS, with rapid early events and survival curves separating within the first 250 days from diagnosis. This observation is grounded in the biological context provided by the broader rare cell landscape, but it is the D cell signal, not the vimentin-positive signal, that carries the primary clinical message from this dataset.

The D|V (Vimentin^+^/CD45^−^) population provided the strongest statistical discrimination between OS patients and healthy donors (*p* = 4.64 × 10^−12^), consistent with the mesenchymal identity of OS. However, vimentin is expressed not only by mesenchymal tumor cells but also by a range of non-neoplastic circulating cells including activated monocytes, endothelial progenitors, and stromal-derived cells that may be present in healthy adult peripheral blood. The D|V signal, while significantly elevated in OS relative to this adult baseline, cannot be interpreted as tumor-specific without further orthogonal validation. Critically, D|V count at a single overt-disease timepoint did not stratify RFS within the OS cohort (log-rank *p* = 0.8367), confirming that discrimination from healthy donors and prognostic stratification within OS represent distinct analytical questions requiring distinct analytes.

The biological identity of D cells warrants careful consideration. DAPI-only classification indicates that these events are morphologically distinct from leukocytes and do not express any of the four immunophenotypic channel markers, but it does not imply biological simplicity. Several interpretations are plausible: D cells may represent circulating OS cells that have downregulated all four channel markers during hematogenous transit; they may express vimentin at the cell surface (CSV) in a form inaccessible to the permeabilization-based intracellular staining protocol used here; or they may constitute a non-neoplastic cell type whose abundance is modulated by tumor-driven inflammatory or paracrine signaling. A related, non-exclusive possibility is that D cells reflect a host response to tumor burden (for example, circulating stromal or endothelial-breakdown products released by an actively growing or vascularly disrupted tumor) rather than a directly malignant population; the present data cannot distinguish this possibility from direct tumor derivation, and targeted single-cell proteomic profiling is required to resolve it. Regardless of identity, their consistent correlation with metastatic burden, disease subgroup, and time to relapse argues for biological encoding of disease aggressiveness that is not redundant with the D|V signal.

Across all three levels of genomic interrogation (tumor tissue, plasma cfDNA, and single circulating rare cells), the data converge on a consistent finding: DNA-based analyses in this cohort did not generate prognostically actionable signal, while the circulating rare cell phenotypic landscape did. Tumor tissue sequencing identified a genomically heterogeneous cohort with TP53 as the most frequent alteration (38%; 8/21 patients by targeted panel), associating with disease stage. The lower observed frequency relative to the >90% reported by comprehensive genomic profiling reflects the inherent limitations of hotspot-based targeted panels in capturing the structural TP53 events (deletions, rearrangements, and fusions) that predominate in OS genomics. Nonetheless, TP53 alterations in this cohort associated with disease stage, and the fact that 33.3% of patients harbored no clinically significant variants is consistent with the inadequacy of mutation-centric panels for a structurally complex tumor [5]. Plasma cfDNA was similarly uninformative at scale: 78.6% of samples fell at or below the 3% detection limit, with fragment size analysis confirming that sample quality was non-limiting [6,7]. Single-cell CNA profiling across five patients revealed predominantly neutral profiles with no clonal alterations. We note that both the ULP-WGS (5000 genome-wide bins) and scCNA workflows used here are designed for large-scale copy-number changes and are not well powered to detect the smaller focal alterations or complex structural rearrangements that characterize osteosarcoma genomics; the predominantly neutral profiles reported here should therefore be interpreted as uninformative at the resolution tested, not as evidence against tumor-derived genomic alteration in these cells and samples. The most parsimonious interpretation is that the prognostic information carried by D cells resides in their morphometric and proteomic properties rather than their DNA, motivating multiplexed single-cell proteomic characterization as the primary next analytical step. Importantly, the predominantly neutral scCNA profiles observed in D cells do not exclude a tumor relationship; rather, these cells may represent non-neoplastic circulating cells whose abundance is modulated by tumor-driven paracrine or inflammatory signaling. Their prognostic relevance may therefore reflect systemic tumor biology rather than direct clonal dissemination, a distinction that single-cell proteomic characterization is better positioned to resolve than DNA sequencing.

This study’s enrichment-free architecture differs from prior sarcoma CTC work in one critical respect: the absence of any pre-analytic selection step. Size-based filtration [10], CSV-based immunoaffinity capture [11,12], and ganglioside-targeting microfluidic devices [13] each impose a biological selection criterion that may exclude populations lacking the targeted property, including potentially the D cell population identified here as prognostically relevant. Whether enrichment-based and enrichment-free platforms recover complementary or overlapping rare cell populations, and whether the D cell survival signal is reproducible with independent detection methods, are important questions for future comparative studies.

Several limitations require acknowledgment. Osteosarcoma is rare (our referral center enrolls approximately 3–4 newly diagnosed patients per year), and while the present cohort (*n* = 25 patients; *n* = 19 with sufficient follow-up for survival analysis) represents a substantial multi-year, single-institution collection effort for this tumor type, it remains underpowered for multivariable modeling and cannot support a validated prognostic claim; all findings reported here should be regarded as exploratory and hypothesis-generating, warranting confirmation in a larger, independent, multi-institutional cohort. The healthy donor (ND) comparator group consisted of adults, whereas the OS cohort was pediatric and young adult (median age 15); pediatric healthy-donor peripheral blood is exceptionally difficult to obtain, and although our IRB protocol permits collection of a healthy sibling control sample, we have not yet succeeded in enrolling one. Age-related differences in baseline immune and stromal rare-cell populations and bone turnover dynamics likely contribute to the OS-vs-ND signal reported here, and the ND comparison should be interpreted as establishing detectability above an adult peripheral blood background rather than age-matched specificity. The data-driven D cell cutpoint introduces optimization bias and must be validated in independent data using bootstrap or cross-validated approaches. The biological identity of D cells has not been confirmed through orthogonal single-cell proteomic or genomic validation; targeted proteomic profiling of this population is planned as the highest-priority next step. No disease-control cohort was included, precluding specificity assessment relative to other mesenchymal malignancies. Longitudinal data from over 150 samples across the disease course in 29 patients remain to be analyzed, representing the most important near-term analytical investment for establishing whether D cell dynamics track disease trajectory. We have not added new patients or samples in this revision; we consider prospective validation in an independent, larger cohort the appropriate next study rather than an expansion of the present exploratory dataset.

## 5. Conclusions

Enrichment-free circulating rare cell profiling in pediatric OS identifies a DAPI-only cell population whose abundance at overt disease timepoints significantly stratifies relapse-free survival (log-rank *p* = 0.0003), with high D cell burden associating with markedly shorter relapse-free intervals, as well as with metastatic burden, time to relapse, and disease subgroup. This population is identifiable by nuclear morphology alone, without biomarker enrichment. The Vimentin^+^/CD45^−^ population provides the strongest discrimination of OS patients from healthy donors but does not carry independent prognostic information within the OS cohort, underscoring the distinction between disease presence and disease aggressiveness as liquid biopsy endpoints. Genomic analyses at the plasma, single-cell, and tumor tissue levels were uniformly uninformative for prognosis, reinforcing the primacy of cell-based phenotypic and morphometric characterization in this disease. This is, to our knowledge, the first report of an enrichment-free circulating rare cell association with relapse-free survival in osteosarcoma, and we present it as a novel, hypothesis-generating finding rather than a validated clinical tool. Prospective validation of DAPI-only rare cell enumeration in a larger, independent cohort, alongside single-cell proteomic characterization of this population, represents the appropriate next step toward determining whether this approach can eventually inform liquid biopsy strategies for pediatric osteosarcoma.

## Figures and Tables

**Figure 1:**
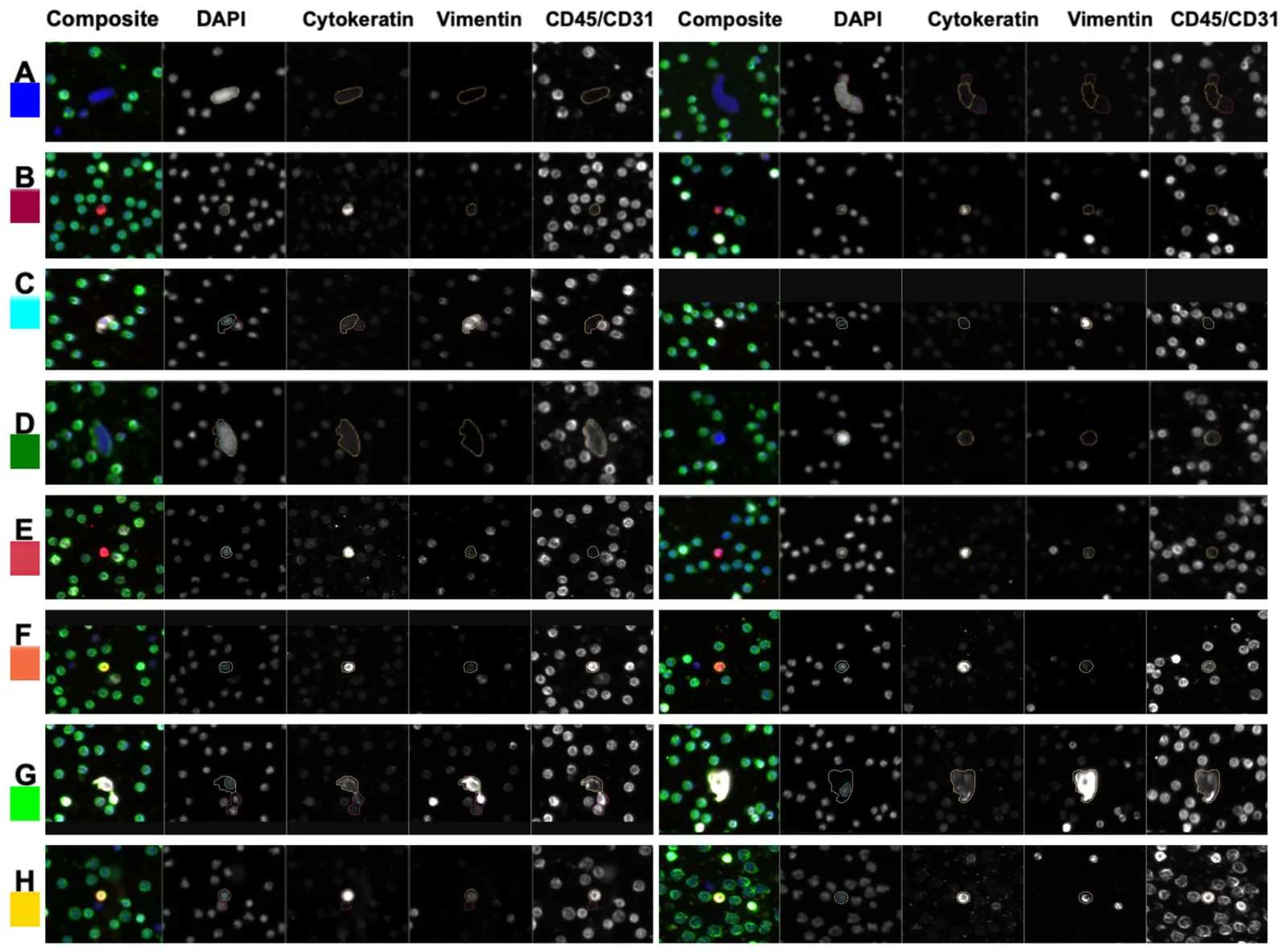
Representative circulating rare cell gallery from OS patient peripheral blood. Images taken at 100× magnification. Composite immunofluorescence images show individual rare cell events (centered within the image) across all eight phenotypic classification categories ((**A**–**H**), two examples per row), identified by combinations of fluorescent markers. Each cell is displayed across five channels: composite overlay, DAPI (nuclear, grayscale; blue in composite), cytokeratin (Alexa Fluor 555; red), vimentin (Alexa Fluor 488; white), and CD45/CD31 (Alexa Fluor 647; green). Cell outlines indicated by orange contours visible across channels. Rows: (**A**) D (DAPI/CK-/V-/CD-, large morphology); (**B**) D|CK (DAPI/CK/V-/CD-); (**C**) D|V (DAPI/V/CD-); (**D**) D|CD (DAPI/CD); (**E**) D|CK|V (DAPI/CK/V); (**F**) D|CK|CD (DAPI/CK/CD); (**G**) D|V|CD (DAPI/V/CD); (**H**) D|CK|V|CD (DAPI/CK/V/CD).

**Figure 2:**
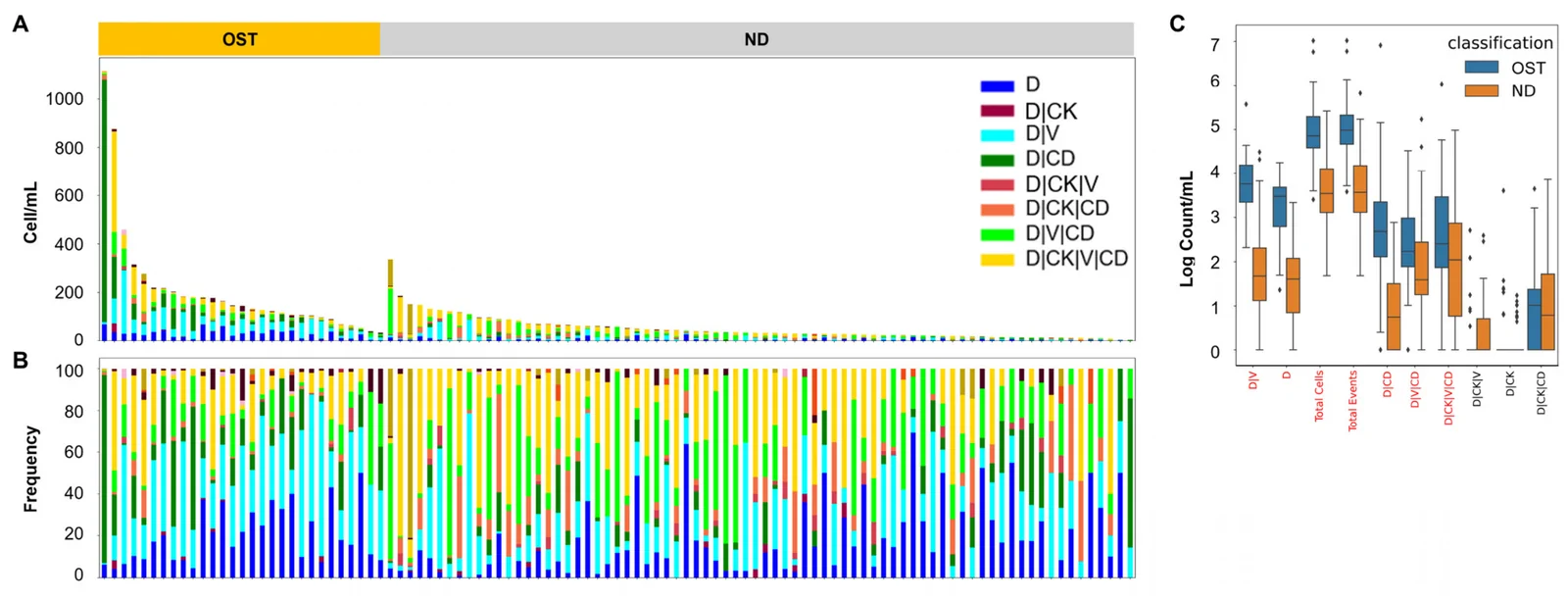
Circulating rare event profiles in OS patients compared with healthy donors (ND). (**A**) Absolute enumeration of each rare event category per sample (cells/mL), ordered by total count. OS samples (orange header bar) are separated from ND samples (gray header bar). (**B**) Sample-level frequency composition of each rare event category, with the D population listed first followed by D|V as the two populations of primary interest. (**C**) Log-transformed box-and-whisker plots of rare events per mL; OS (blue) versus ND (orange). Phenotypes reaching statistical significance (Wilcoxon rank-sum, *p* < 0.05) are labeled in red along the x-axis, ordered left to right by ascending *p*-value. All events were identified by automated OCULAR detection followed by manual data curation. OS: *n* = 29 samples from 25 patients; ND: *n* = 76. Points represent outliers.

**Figure 3:**
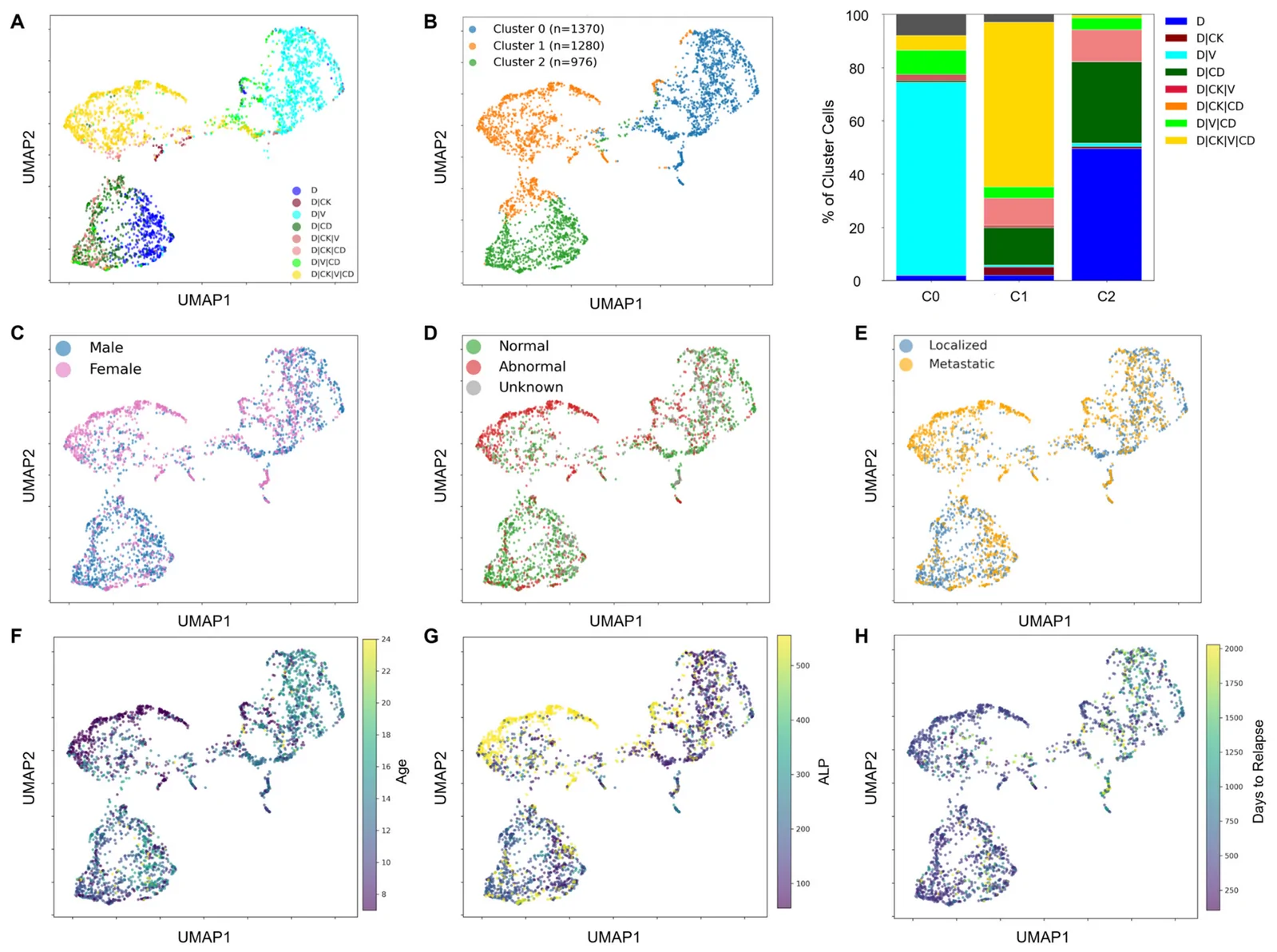
Cell-level UMAP embedding of 3626 circulating rare cells based on full morphometric feature space (Z-normalized embeddings). All events were identified by automated OCULAR detection followed by manual data curation. Top row: UMAP colored by (**A**) biomarker-channel classification (D, D|CK, D|V, D|CD, D|CK|V, D|CK|CD, D|CK|V|CD, D|V|CD as shown in legend); (**B**) Leiden clustering (**left**) and cluster composition (**right**); (**C**) sex (Male = blue, Female = pink); (**D**) lactate dehydrogenase (LDH, Normal = green, Abnormal = red, Unknown = grey); (**E**) stage (Localized = blue, Metastatic = orange); (**F**) Age (years); (**G**) alkaline phosphatase (ALP, U/L); (**H**) days to relapse.

**Figure 4:**
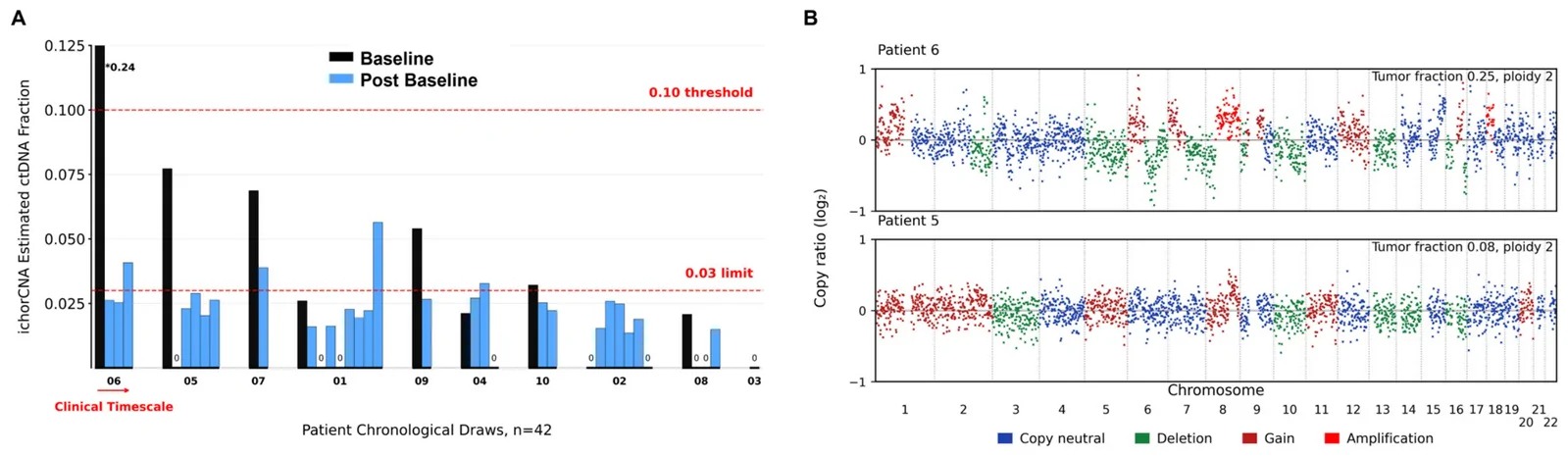
ichorCNA tumor fraction estimation from OS patient plasma samples (*n* = 42). (**A**) Bar plot of cfDNA ULP-WGS tumor fraction across all unique blood draws, patients are grouped then ordered by decreasing tumor fraction. Bars are color-coded: black (baseline draws) and blue (post baseline draws. Sample identifiers have been omitted from the x-axis for clarity, post baseline samples can include post-surgery, post-treatment, between treatment cycles and end of therapy conditions. Dashed red lines mark the 10% confidence threshold and 3% detection limit. (**B**) Representative genome-wide copy number profiles from the two highest-confidence samples. Copy number ratio (log_2_) across autosomes per ichorCNA pipeline output: Red = amplification; Dark red = gain; Blue = neutral; Green = heterozygous deletion. Top panel: Patient 6, TF = 0.246, ploidy 2. Bottom panel: Patient 5, TF = 0.077, ploidy 2.

**Figure 5:**
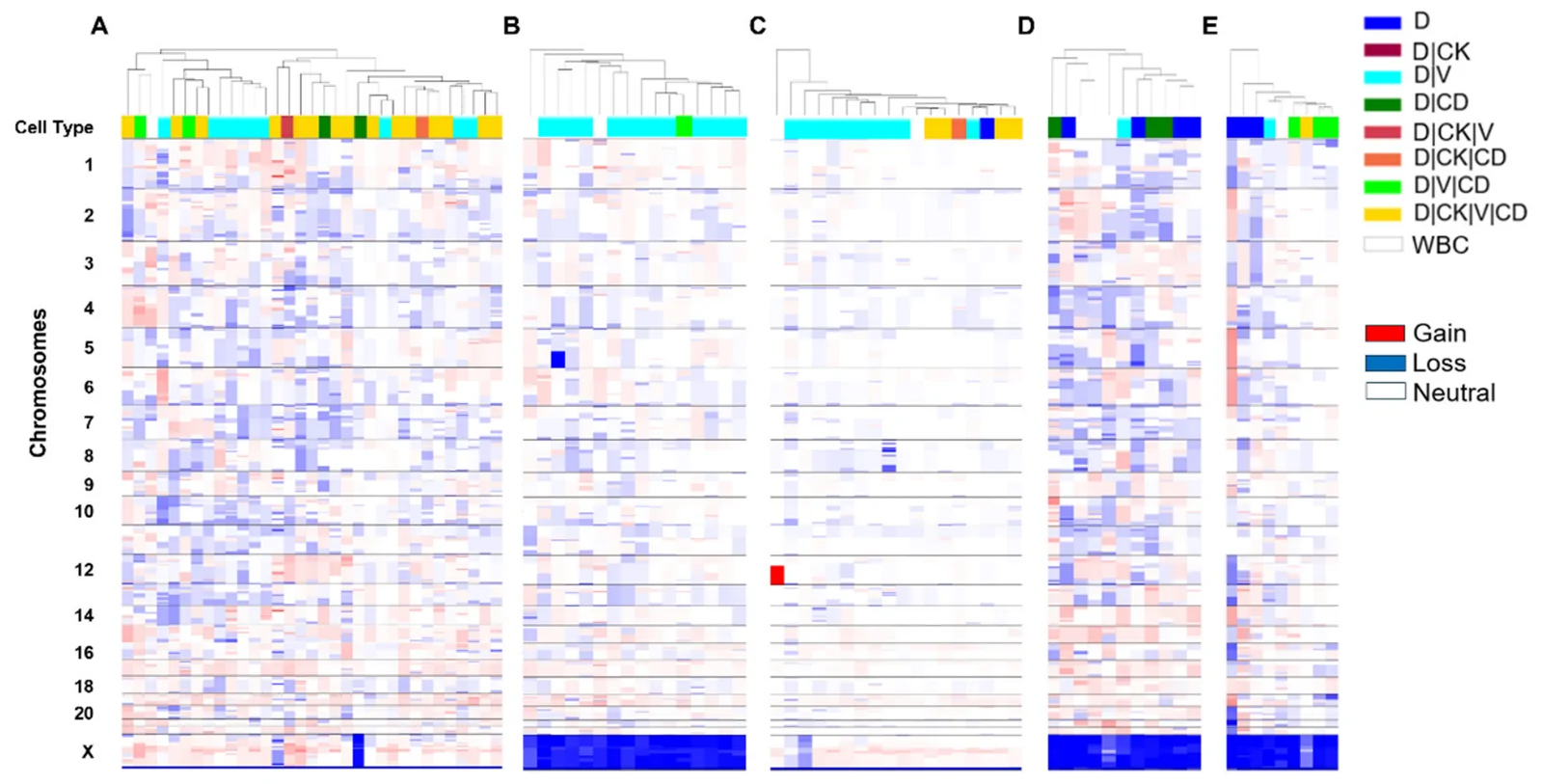
Single-cell CNA profiles for circulating rare cells from five OS patients. (**A**) Patient 17; (**B**) Patient 18; (**C**) Patient 22; (**D**) Patient 24 (relapse); (**E**) Patient 4 (progression). Heatmap columns represent individual cells passing the 400,000 mapped bin threshold (80 rare cells, 9 WBC controls); rows correspond to chromosomal positions (chromosomes 1–22, X labeled on y-axis). Cell type annotation bars are shown above each heatmap, color-coded by immunophenotypic classification: D (dark blue), D|CK (dark red), D|V (cyan), D|CD (dark green), D|CK|V (pink), D|CK|CD (orange), D|V|CD (lime green), D|CK|V|CD (yellow), WBC (white). Copy number state: gain (red), neutral (white/pale), loss (blue). The color scale is calibrated such that values of 1.5 and 0.5 represent a gain or loss of one copy in a diploid cell; pale colors reflect less than a full copy change. One focal full loss is visible in panel (**B**) (chromosome 5) and one focal full gain in panel (**C**) (chromosome 12). Predominantly neutral profiles are observed across all five patients; no shared clonal alterations meet the clonality criterion. Note that cells labeled WBC were profiled from the WBC fraction but displayed non-flat copy number profiles; these cells are retained for transparency. DAPI-only (**D**) and D|CD cells showed the highest mean per-cell genomic instability scores (182.3 and 120.6, respectively); D|V cells showed intermediate instability (mean 72.1). One-way ANOVA across classification categories: F(6,73) = 3.67, η^2^ = 0.232, *p* = 0.0031.

**Figure 6:**
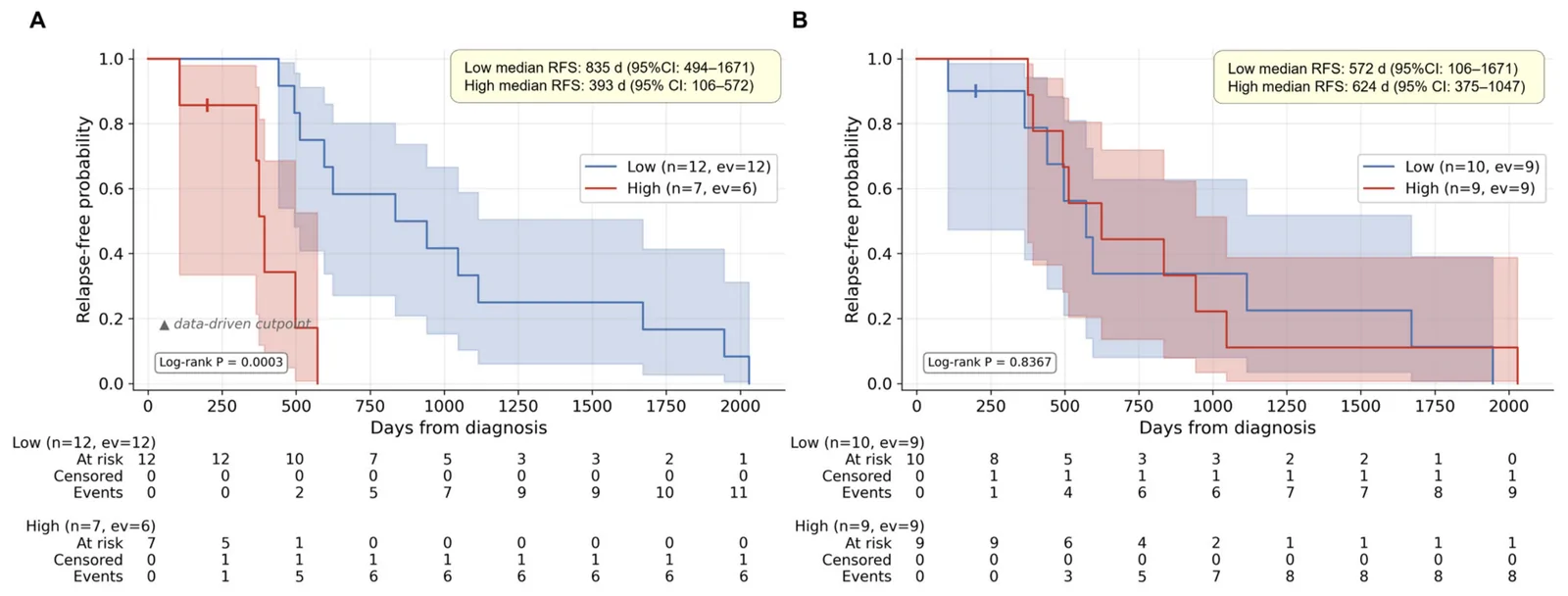
Kaplan-Meier relapse-free survival analysis stratified by circulating rare cell count at overt disease timepoints in pediatric OS patients with sufficient follow-up (*n* = 19; 6 patients excluded due to insufficient follow-up time). (**A**) DAPI-only (D) cells, dichotomized by data-driven optimal cutpoint (38.32 cells/mL; triangle marker): high (red; *n* = 7, relapse events = 6) vs. low (blue; *n* = 12, relapse events = 12). Log-rank *p* = 0.0003 (naive). High D cell burden is associated with significantly shorter RFS; this association remained significant after permutation testing (*p* = 0.027) and bootstrap optimism correction (*p* = 0.003; see Section 3.6). (**B**) D|V (Vimentin^+^/CD45^−^) cells, dichotomized at the cohort median (38.54 cells/mL): high (red; *n* = 9, relapse events = 9) vs. low (blue; *n* = 10, relapse events = 9). Log-rank *p* = 0.8367. D|V count does not stratify RFS despite being the strongest OS-vs-ND discriminator. Shaded bands indicate 95% confidence intervals. X-axis: days from diagnosis. All analyses are exploratory.

**Table 1:** Patient Characteristics (*n* = 25).

Age (Years)	
Median	13
Range	7–24
Gender	
Male	15 (60%)
Female	10 (40%)
Ethnicity	
Hispanic	17 (68%)
Non-Hispanic	8 (32%)
Stage at Diagnosis	
Localized	14 (56%)
Metastatic	11 (44%)
Disease Status at Study Enrollment	
Newly Diagnosed	11 (44%)
Relapse	14 (56%)
Serum LDH at Study Enrollment	
Normal	14 (56%)
Abnormal	5 (20%)
Not available	6 (24%)
Serum ALP at Enrollment	
Normal	17 (68%)
Abnormal	7 (28%)
Not available	1 (4%)

**Table 2:** Rare cell phenotypes reaching statistical significance in OS patients vs. healthy donors (clinically apparent disease timepoints). D  =  DAPI; V  =  vimentin; CK  =  cytokeratin; CD  =  CD45/CD31. Two-sided Wilcoxon rank-sum test; *p*  <  0.05. Benjamini-Hochberg false discovery rate calculated for 10 comparisons.

Rare Cell Phenotype	OS Median (Range), Cells/mL	ND Median (Range), Cells/mL	*p* Value	Q Value
D|V	41.96 (9–261)	4.35 (0–88)	4.64 × 10^−12^	4.64 × 10^−11^
D	31.46 (3–69)	4 (0–27)	1.88 × 10^−11^	9.37 × 10^−11^
Total Cells	127.26 (29–1114)	33.57 (4–225)	2.60 × 10^−10^	8.65 × 10^−10^
D|CD	13.63 (0–1004)	1.11 (0–17)	1.16 × 10^−8^	2.91 × 10^−8^
D|V|CD	8.34 (0–91)	3.92 (0–186)	0.011	0.022
D|CK|V|CD	10.09 (0–415)	6.7 (0–145)	0.033	0.055
Total CK+ Cells	17.16 (0–462)	9.96 (0–170)	0.046	0.066

**Table 3:** Significant associations between tumor tissue genomic variants and rare cell phenotype variables. Two-sided Wilcoxon rank-sum test or Fisher’s exact test; *p* < 0.05. Benjamini-Hochberg false discovery rate calculated for 80 comparisons.

Tissue Genomic Variant	Associated Metric	*p* Value	Q Value
CCND3 (14%)	D|V	0.002	0.073
CCND3	D|CK|V	0.005	0.102
CDK4 (10%)	D|CK|V	0.002	0.073
CDK4	D|V	0.003	0.073
PIK3CA (5%)	D	0.044	0.708

**Table 4:** Significant associations between circulating rare cell phenotypes and clinical variables. Statistical approach denotes the method used for each comparison: Spearman rank correlation for continuous-continuous, or two-sided Wilcoxon rank-sum test for categorical-continuous; *p* < 0.05. Benjamini-Hochberg false discovery rate calculated for 140 comparisons.

Clinical Variable (Frequency; Median, Range)	Rare Cell Phenotype	Statistical Approach	*p* Value	Q Value
Days to Relapse (583.5, 106–2030)	D	Spearman	0.005	0.443
Lung metastasis at diagnosis (44%)	D	Wilcoxon	0.006	0.443
Bone metastasis at diagnosis (20%)	D	Wilcoxon	0.015	0.625
Days to Relapse	Total Cells	Spearman	0.020	0.625
Distant Relapse (52%)	D|CK|CD	Wilcoxon	0.022	0.625
Patient Relapse (80%)	D|V	Wilcoxon	0.031	0.729
Distant Relapse	Total Cells	Wilcoxon	0.050	0.803

## Data Availability

All data discussed in this manuscript are included in the main manuscript text or Supplementary Materials. The imaging data are available through the BLOODPAC Portal, Accession ID “BPPDS-0000002” (https://portal.bloodpac.org/data/BPPDS-0000002, accessed on 23 September 2026).

## Supplementary Materials

The following supporting information can be downloaded at: https://www.mdpi.com/article/10.3390/cancers18193123/s1. Figure S1. Sample allocation flow diagram for the osteosarcoma liquid biopsy cohort. Of 25 pediatric osteosarcoma patients included in this study, 21 had matched tumor tissue genomic data available via the OncoKids^®^ Cancer Panel. A total of 61 blood samples were collected across the cohort. Samples were allocated to two analytic streams: 29 samples drawn at clinically apparent disease timepoints (24 pre-treatment and 5 at disease progression) were used for enrichment-free circulating rare cell analysis; 32 samples collected at alternative timepoints were used for cell-free DNA (cfDNA) analysis. An additional 10 samples from the clinically apparent disease timepoint cohort were shared with the cfDNA analysis stream, yielding a total of 42 samples for ichorCNA-based ultra-low-pass whole-genome sequencing. The circulating rare cell and cfDNA datasets are otherwise non-overlapping. Figure S2. Patient-level distribution of circulating rare cells in UMAP embedding space. (A) UMAP projection of 3626 circulating rare cells from 25 osteosarcoma patients, colored by patient identifier and collection timepoint. Each point represents a single rare cell event; cell counts per sample are indicated in the legend (*n* = total events per patient). The embedding captures the full morphometric and immunophenotypic diversity of the cohort, with cells distributed across the three Leiden clusters described in Figure 3. (B–D) Individual patient contributions to the UMAP highlighted for the three highest-contributing patients. (B) CHLA-OS0013 (727 cells, 20.0% of total events), whose cells predominate in Cluster 1 (the most heterogeneous cluster with mixed phenotypic composition including D|V|CD, D|CK|V|CD, D|CK|CD, and D|V populations). (C) CHLA-OS0006 (348 cells, 9.6% of total events), whose cells are concentrated in Cluster 2 (the lower UMAP region dominated by the D|CD (CD45/CD31^+^) population). (D) CHLA-OS0026 (235 cells, 6.5% of total events), whose cells predominate in Cluster 0 (the mesenchymal-forward cluster composed primarily of D|V and D|CD cells). Figure S3. Cell-level UMAP embedding of 8055 circulating rare cells based on full morphometric feature space (Z-normalized embeddings) from ND and OS patient samples. All events were identified by automated OCULAR detection followed by manual data curation. UMAP colored by (A) biomarker-channel classification; (B) cohort; (C) 29 OS patient samples; (D) 76 ND samples. Figure S4. ULP-WGS cell-free DNA fragment size distribution from OS patient plasma samples (*n* = 42). (A) Trace data cfDNA ULP-WGS reads across all unique blood draws and subsequent plasma isolation. The traces are labeled with ichorCNA result, red for failing (<3% TumorFx) and green for passing (≥3% TumorFx). (B) Bar plot quantification of reads versus fragment size compared between passing (*n* = 9) and failing (*n* = 33) ichorCNA samples. Group differences were assessed by two-sided Mann-Whitney U tests with Benjamini-Hochberg correction; no comparisons were significant (FDR-adjusted *p* > 0.05). Short is number of reads with size range 90–150 bp (*p*-value = 0.208, FDR = 0.356), Mononucleosomal is 140–190 bp (*p*-value = 0.231, FDR = 0.356) and Dinucleosomal is 300–380 bp (*p*-value = 0.22, FDR = 0.356). Figure S5. DAPI-only (D) circulating rare cell count versus plasma ichorCNA tumor fraction at matched baseline timepoints, for the 10 patients with both analytes available. Each point represents one patient, colored by clinical stage at diagnosis (localized, teal; metastatic, orange). Solid line shows the linear fit. D-cell count and tumor fraction were positively correlated (Pearson r = 0.61, R^2^ = 0.37, 95% CI −0.04 to 0.89) but the association did not reach statistical significance at this sample size (*p* = 0.064). Figure S6. Representative single-cell genomic and immunofluorescence profile of a WBC-classified circulating rare cell (Patient 17). (A) Genome-wide copy number profile displayed as ratio-to-median across chromosomes 1–22, X, and Y. Gray dots represent individual bin-level copy number ratios; the black segmentation line indicates the piecewise constant fit. A diploid leukocyte control would be expected to produce a flat profile centered at 1.0; this cell displays widespread segmental copy number alterations inconsistent with a diploid baseline, with a GI score of 142.4. (B) Corresponding montage images of the same cell at 100× magnification: composite overlay (left), DAPI (nuclear), cytokeratin, vimentin, and CD45/CD31. The cell outline is indicated by an orange contour. The immunofluorescence profile is consistent with a WBC morphology (small nuclear size, rounded morphology, and CD45/CD31 channel positivity) supporting its classification as a leukocyte despite the unstable genomic profile. The discordance between IF-based WBC classification and the non-diploid CNA profile is discussed in Section 3.3. Figure S7. Overall survival, median-split sensitivity analysis in the same 19 pediatric OS patients with sufficient follow-up as in Figure 6. (A) DAPI-only (D) cells, dichotomized at the cohort median (31.48 cells/mL): low (blue; *n* = 9, events = 1) vs. high (red; *n* = 10, events = 6). Low-group median OS was not reached; high-group median OS was 2373 days (log-rank *p* = 0.0565). (B) D|V (Vimentin^+^/CD45^−^) cells, dichotomized at the cohort median (38.54 cells/mL): low (blue; *n* = 9, events = 2) vs. high (red; *n* = 10, events = 5). Median OS was 2702 days (low) vs. 1331 days (high; log-rank *p* = 0.0782). Shaded bands indicate 95% confidence intervals; risk tables below each panel show numbers at risk, censored, and events over time. As in Figure 6, group assignment here uses a cohort-median split rather than the data-driven optimal cutpoint used for the primary D cell RFS analysis, provided as a sensitivity analysis using overall survival as the endpoint. Both comparisons trend in the same direction as the corresponding RFS analyses but do not reach significance at this sample size.

## Abbreviations

ALP: alkaline phosphatase; ANOVA: analysis of variance; CK: cytokeratin; CNA: copy number alteration; CSV: cell surface vimentin; CTC: circulating tumor cell; cfDNA: cell-free DNA; DAPI: 4′,6-diamidino-2-phenylindole; EpCAM: epithelial cell adhesion molecule; FDR: false discovery rate; GI: genomic instability; ichorCNA: inferring cancer history from off-target reads; LDH: lactate dehydrogenase; ND: healthy donor; OCULAR: Outlier Clustering Unsupervised Learning Automated Report; OS: osteosarcoma; RFS: relapse-free survival; scCNA: single-cell copy number alteration; ULP-WGS: ultra-low-pass whole-genome sequencing; UMAP: Uniform Manifold Approximation and Projection; V/Vim: vimentin; WBC: white blood cell; WGA: whole-genome amplification.
