# Supplementary material for: Enrichment-Free Rare Cell Profiling in Pediatric Osteosarcoma Reveals a Circulating Cell Population Associated with Relapse-Free Survival
Source: Cancers (Basel). 2026 Sep 26;18(19):3123. doi: 10.3390/cancers18193123 (PMC13663973; doi:10.3390/cancers18193123)
Supplement: Supplementary file 1 [file cancers-18-03123-s001.zip › cancers-4497797-supplementary.pdf]

Supplemental Information

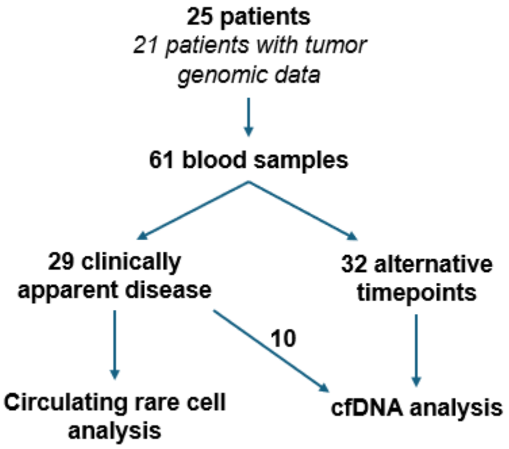

Supplemental Figure S1. Sample allocation flow diagram for the osteosarcoma liquid biopsy cohort. Of 25 pediatric osteosarcoma patients enrolled, 21 had matched tumor tissue genomic data available via the OncoKids® Cancer Panel. A total of 61 blood samples were collected across the cohort. Samples were allocated to two analytic streams: 29 samples drawn at clinically apparent disease timepoints (24 pre-treatment and 5 at disease progression) were used for enrichment-free circulating rare cell analysis; 32 samples collected at alternative timepoints were used for cell-free DNA (cfDNA) analysis. An additional 10 samples from the clinically apparent disease timepoint cohort were shared with the cfDNA analysis stream, yielding a total of 42 samples for ichorCNA-based ultra-low-pass whole-genome sequencing. The circulating rare cell and cfDNA datasets are otherwise non-overlapping.

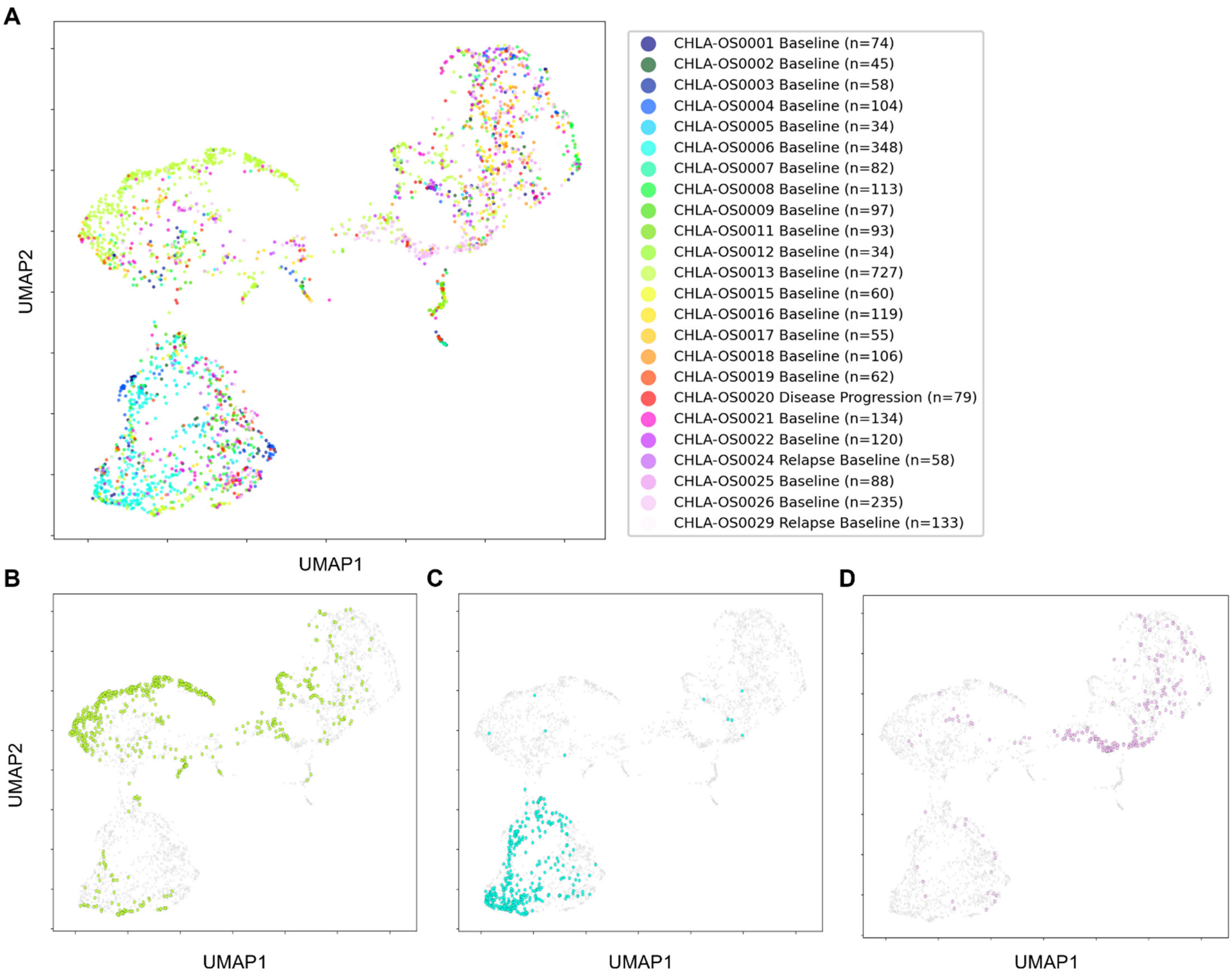

Supplemental Figure S2. Patient-level distribution of circulating rare cells in UMAP embedding space. A) UMAP projection of 3,626 circulating rare cells from 25 osteosarcoma patients, colored by patient identifier and collection timepoint. Each point represents a single rare cell event; cell counts per sample are indicated in the legend (n = total events per patient). The embedding captures the full morphometric and immunophenotypic diversity of the cohort, with cells distributed across the three Leiden clusters described in Figure 3. B–D) Individual patient contributions to the UMAP highlighted for the three highest-contributing patients. B) CHLA-OS0013 (727 cells, 20.0% of total events), whose cells predominate in Cluster 1 — the most heterogeneous cluster with mixed phenotypic composition including D|V|CD, D|CK|V|CD, D|CK|CD, and D|V populations. C) CHLA-OS0006 (348 cells, 9.6% of total events), whose cells are concentrated in Cluster 2 — the lower UMAP region dominated by the D|CD (CD45/CD31^{+}) population. D) CHLA-OS0026 (235 cells, 6.5% of total events), whose cells predominate in Cluster 0 — the mesenchymal-forward cluster composed primarily of D|V and D|CD cells.

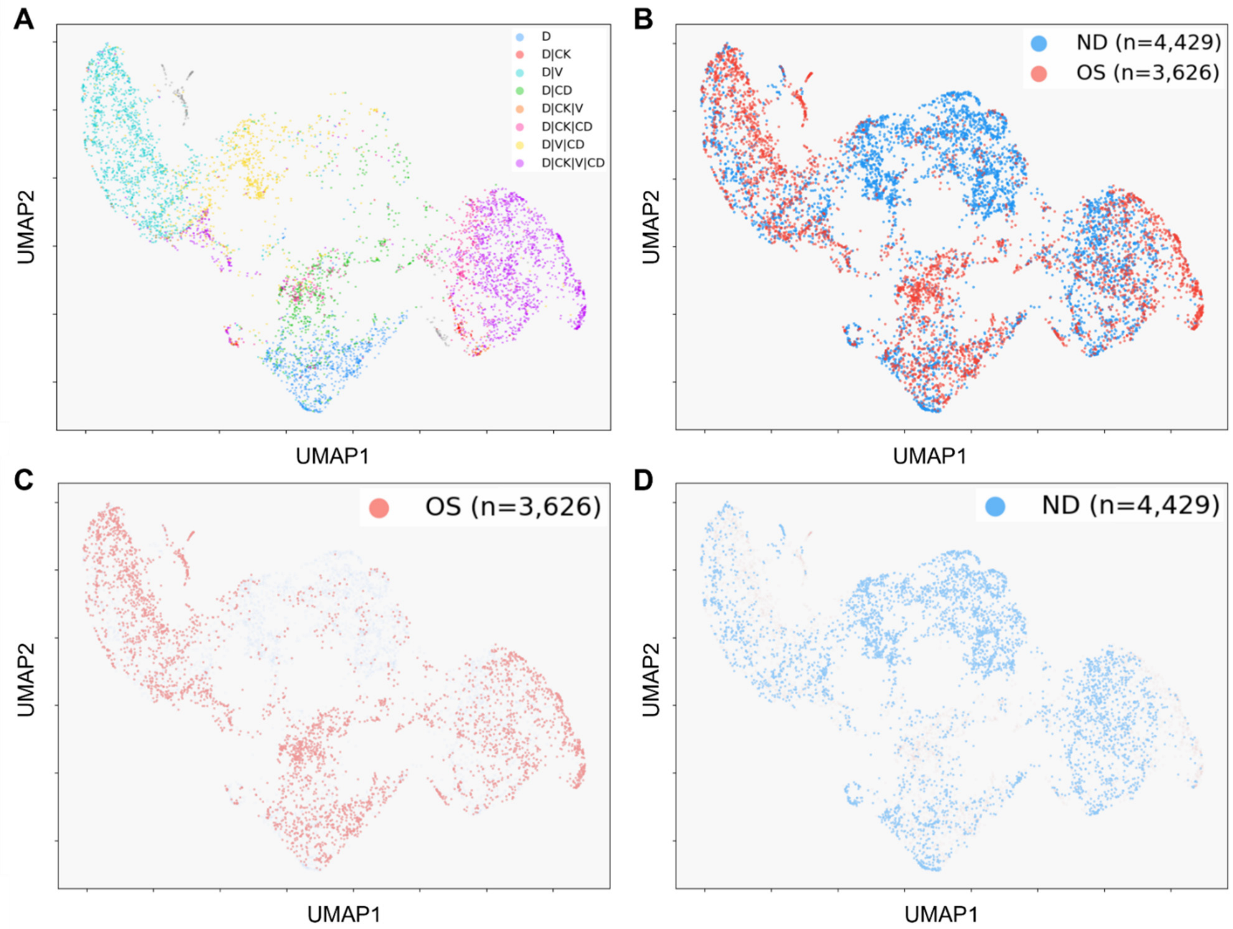

Supplemental Figure S3. Cell-level UMAP embedding of 8,055 circulating rare cells based on full morphometric feature space (Z-normalized embeddings) from ND and OS patient samples. All events were identified by automated OCULAR detection followed by manual data curation. UMAP colored by A) biomarker-channel classification; B) cohort; C) 29 OS patient samples; D) 76 ND samples.

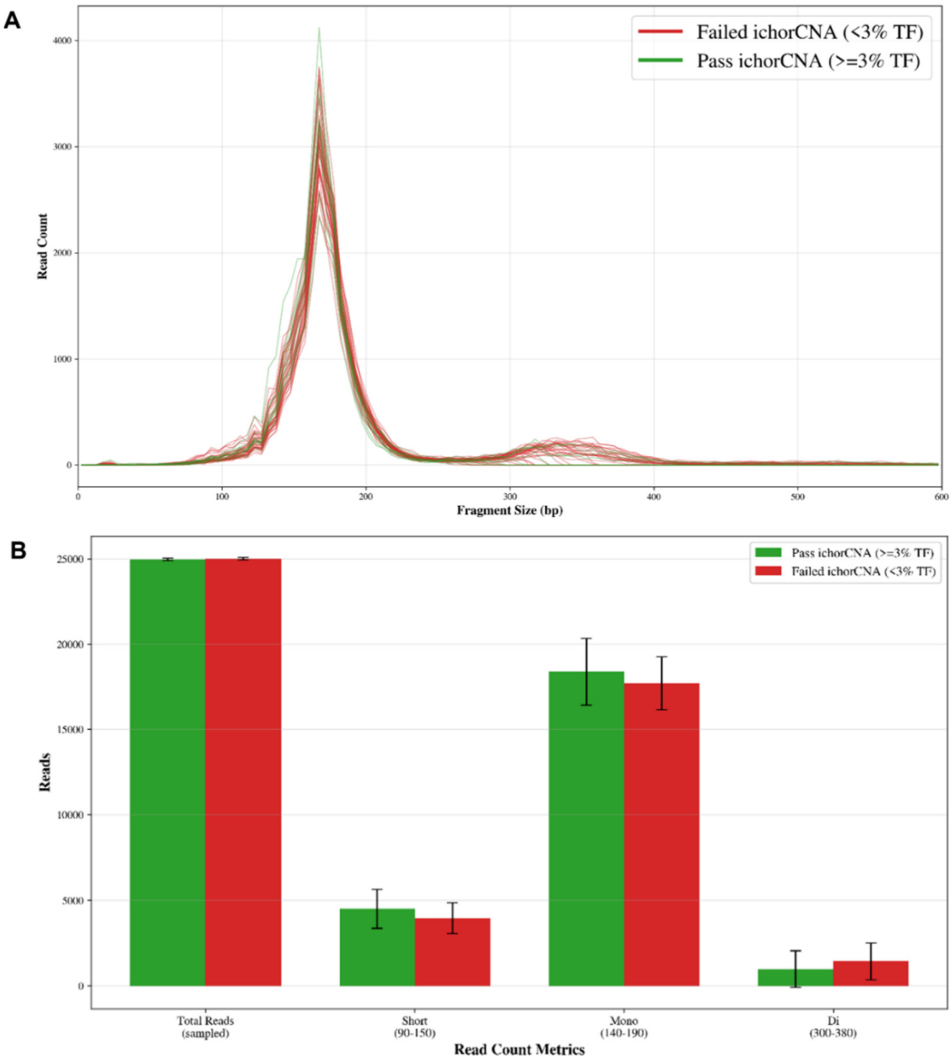

Supplemental Figure S4. ULP-WGS cell-free DNA fragment size distribution from OS patient plasma samples (n=42). A) Trace data cfDNA ULP-WGS reads across all unique blood draws and subsequent plasma isolation. The traces are labeled with ichorCNA result, red for failing (<3% TumorFx) and green for passing (≥ 3% TumorFx). B) Bar plot quantification of reads versus fragment size compared between passing (n=9) and failing (n=33) ichorCNA samples. Group differences were assessed by two-sided Mann-Whitney U tests with Benjamini-Hochberg correction; no comparisons were significant (FDR-adjusted $P > 0.05$). Short is number of reads with size range 90-150bp (p-value = 0.208, FDR = 0.356), Mononucleosomal is 140-190bp (p-value = 0.231, FDR = 0.356) and Dinucleosomal is 300-380bp (p-value = 0.22, FDR = 0.356).

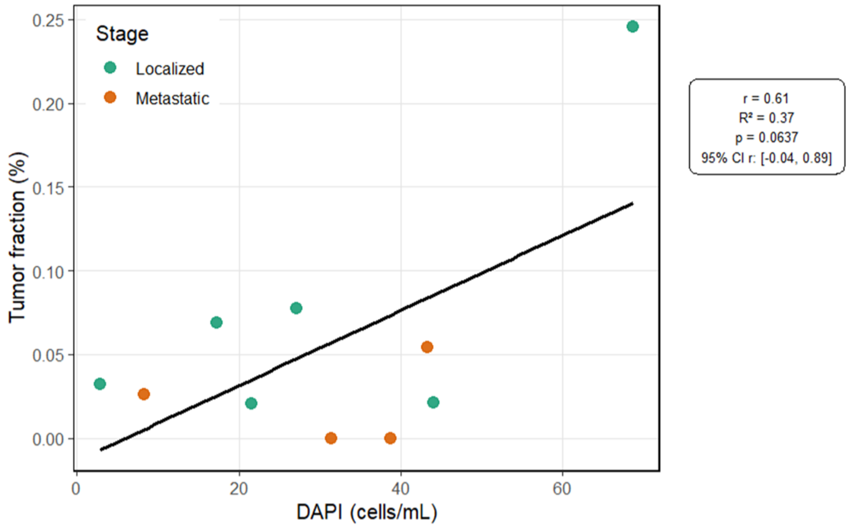

Supplemental Figure S5. DAPI-only (D) circulating rare cell count versus plasma ichorCNA tumor fraction at matched baseline timepoints, for the 10 patients with both analytes available. Each point represents one patient, colored by clinical stage at diagnosis (localized, teal; metastatic, orange). Solid line shows the linear fit. D-cell count and tumor fraction were positively correlated (Pearson r = 0.61, R^2 = 0.37, 95% CI −0.04 to 0.89) but the association did not reach statistical significance at this sample size (P = 0.064).

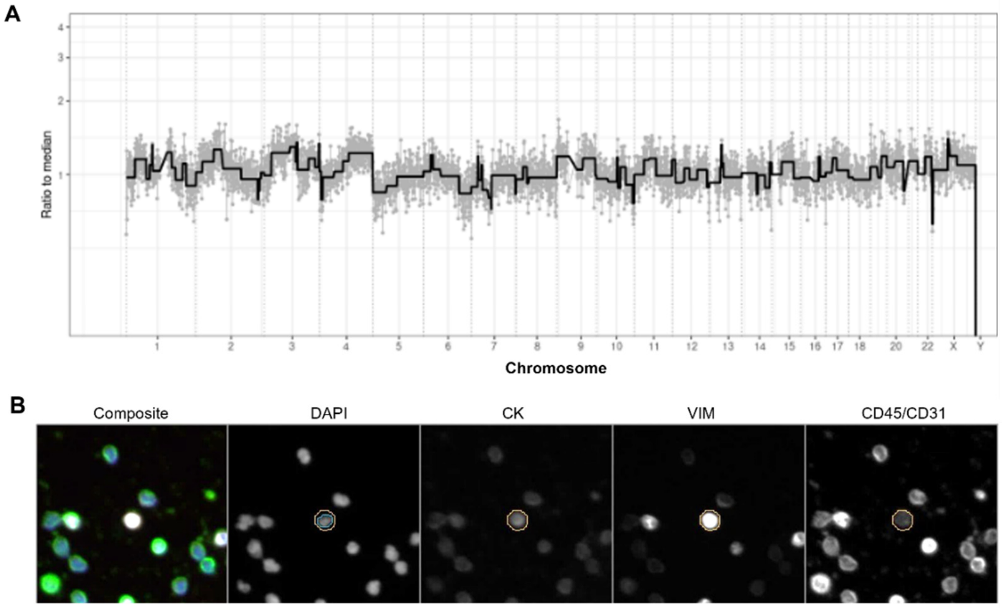

Supplemental Figure S6. Representative single-cell genomic and immunofluorescence profile of a WBC-classified circulating rare cell (Patient 17). A) Genome-wide copy number profile displayed as ratio-to-median across chromosomes 1–22, X, and Y. Gray dots represent individual bin-level copy number ratios; the black segmentation line indicates the piecewise constant fit. A diploid leukocyte control would be expected to produce a flat profile centered at 1.0; this cell displays widespread segmental copy number alterations inconsistent with a diploid baseline, with a GI score of 142.4. B) Corresponding montage images of the same cell at 100× magnification: composite overlay (left), DAPI (nuclear), cytokeratin, vimentin, and CD45/CD31. The cell outline is indicated by an orange contour. The immunofluorescence profile is consistent with a WBC morphology (small nuclear size, rounded morphology, and CD45/CD31 channel positivity) supporting its classification as a leukocyte despite the unstable genomic profile. The discordance between IF-based WBC classification and the non-diploid CNA profile is discussed in Section 3.3.

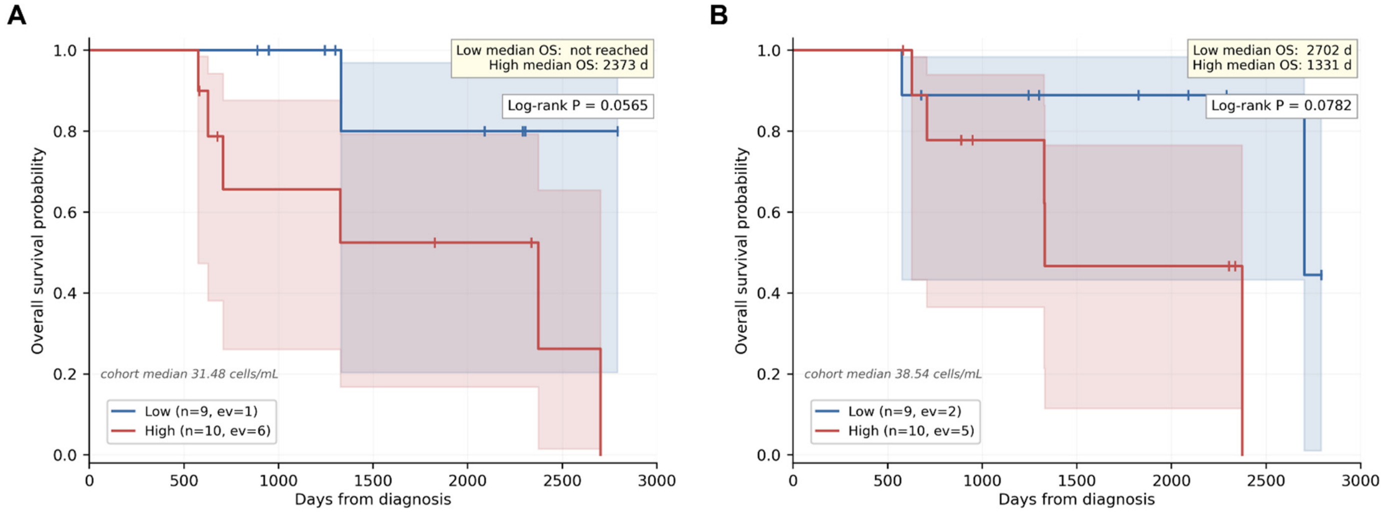

A

Low (n=9, ev=1)							
At risk	9	9	7	4	4	1	0
Censored	0	0	2	4	4	7	8
Events	0	0	0	1	1	1	1

High (n=10, ev=6)							
At risk	10	10	5	4	3	1	0
Censored	0	0	2	2	3	4	4
Events	0	0	3	4	4	5	6

B

Low (n=9, ev=2)							
At risk	9	9	7	5	4	2	0
Censored	0	0	1	3	4	6	7
Events	0	0	1	1	1	1	2

High (n=10, ev=5)							
At risk	10	10	5	3	3	0	0
Censored	0	0	3	3	3	5	5
Events	0	0	2	4	4	5	5

Supplemental Figure S7. Overall survival, median-split sensitivity analysis in the same 19 pediatric OS patients with sufficient follow-up as in Figure 6. A) DAPI-only (D) cells, dichotomized at the cohort median (31.48 cells/mL): low (blue; n = 9, events = 1) vs. high (red; n = 10, events = 6). Low-group median OS was not reached; high-group median OS was 2373 days (log-rank P = 0.0565). B) D|V (Vimentin^{+}/CD45^{-}) cells, dichotomized at the cohort median (38.54 cells/mL): low (blue; n = 9, events = 2) vs. high (red; n = 10, events = 5). Median OS was 2702 days (low) vs. 1331 days (high; log-rank P = 0.0782). Shaded bands indicate 95% confidence intervals; risk tables below each panel show numbers at risk, censored, and events over time. As in Figure 6, group assignment here uses a cohort-median split rather than the data-driven optimal cutpoint used for the primary D cell RFS analysis, provided as a sensitivity analysis using overall survival as the endpoint. Both comparisons trend in the same direction as the corresponding RFS analyses but do not reach significance at this sample size.
